# Genome-wide study of salivary miRNAs identifies miR-423-5p as promising diagnostic and prognostic biomarker in oral squamous cell carcinoma

**DOI:** 10.7150/thno.45157

**Published:** 2021-01-01

**Authors:** Chiara Romani, Elisa Salviato, Alberto Paderno, Laura Zanotti, Antonella Ravaggi, Alberto Deganello, Giulia Berretti, Tommaso Gualtieri, Sergio Marchini, Maurizio D'Incalci, Davide Mattavelli, Cesare Piazza, Paolo Bossi, Chiara Romualdi, Piero Nicolai, Eliana Bignotti

**Affiliations:** 1 Department of Molecular and Translational Medicine, University of Brescia, Brescia, Italy; 2 'Angelo Nocivelli' Institute of Molecular Medicine, University of Brescia and ASST Spedali Civili di Brescia, Brescia, Italy; 3 Department of Biology, University of Padua, via U. Bassi 58/B Padua 35121, Italy; 4 Unit of Otorhinolaryngology - Head and Neck Surgery, ASST Spedali Civili di Brescia, Brescia, Italy; 5 Division of Obstetrics and Gynecology, ASST Spedali Civili di Brescia, Brescia, Italy; 6 Department of Clinical and Experimental Sciences, Division of Obstetrics and Gynecology University of Brescia, Brescia, Italy; 7 Department of Medical and Surgical Specialties, Radiological Sciences and Public Health, University of Brescia, Brescia, Italy; 8 Department of Oncology, IRCCS, "Mario Negri" Institute for Pharmacological Research, Milan, Italy; 9 Department of Otorhinolaryngology, Maxillofacial, and Thyroid Surgery, Fondazione IRCCS, National Cancer Institute of Milan, Milan, Italy; 10 Department of Oncology and Hematoncology, University of Milan, Milan, Italy; 11 Medical Oncology, ASST Spedali Civili di Brescia, Brescia, Italy; 12 CRIBI Biotechnology Center, Viale G Colombo 3, Padua 35131, Italy; 13 Section of Otorhinolaryngology - Head & Neck Surgery, Department of Neurosciences, University of Padua, Italy

**Keywords:** oral squamous cell carcinoma, salivary miRNA, microarray, diagnostic tumor marker, prognostic biomarker

## Abstract

Survival rates of oral squamous cell carcinoma (OSCC) remained substantially unchanged over the last decades; thus, additional prognostic tools are strongly needed. Salivary miRNAs have emerged as excellent non-invasive cancer biomarker candidates, but their association with OSCC prognosis has not been investigated yet. In this study, we analyzed global salivary miRNA expression in OSCC patients and healthy controls, with the aim to define its diagnostic and prognostic potential.

**Methods:** Saliva was collected from patients with newly diagnosed untreated primary OSCC and healthy controls. Global profiling of salivary miRNAs was carried out through a microarray approach, while signature validation was performed by quantitative real-time PCR (RT-qPCR). A stringent statistical approach for microarray and RT-qPCR data normalization was applied. The diagnostic performance of miRNAs and their correlation with OSCC prognosis were comprehensively analyzed.

**Results:** In total, 25 miRNAs emerged as differentially expressed between OSCC patients and healthy controls and, among them, seven were significantly associated with disease-free survival (DFS). miR-106b-5p, miR-423-5p and miR-193b-3p were expressed at high levels in saliva of OSCC patients and their combination displays the best diagnostic performance (ROC - AUC = 0.98). Moreover, high expression of miR-423-5p was an independent predictor of poor DFS, when included in multivariate survival analysis with the number of positive lymph nodes - the only significant clinical prognosticator. Finally, we observed a significant decrease in miR-423-5p expression in matched post-operative saliva samples, suggesting its potential cancer-specific origin.

**Conclusion:** Salivary miRNAs identified in our cohort of patients show to be accurate in OSCC detection and to effectively stratify patients according to their likelihood of relapse. These results, if validated in an independent set of patients, could be particularly promising for screening/follow-up of high-risk populations and useful for preoperative prognostic assessment.

## Introduction

In the last decades, the incidence of oral squamous cell carcinoma (OSCC) has progressively been increasing 1, with approximately 355,000 new cases in 2018 2. At the same time, no major improvement in survival outcomes has been observed. Conventional treatment for locally advanced disease comprises wide surgical resection of the tumor, neck dissection and adjuvant (chemo)-radiotherapy according to pathological staging and risk factors 3. In view of the crucial role of this anatomic site in many physiological conditions, surgery is carefully tailored in order to find the optimal balance between functional and oncologic outcomes. In this regard, the introduction of compartmental approaches 4-7, which can be modulated according to tumor extension, can be considered a significant advancement. However, preoperative evaluation only relies on clinical and radiologic data, without considering tumor biology and microenvironment. Indeed, different studies demonstrated that tumors with similar TNM staging may present wide differences in biologic features having an impact on prognosis (i.e., worst pattern of invasion, tumor budding, tumor-stroma ratio and gene-expression signatures) 8-13. However, the small size of preoperative tumor biopsies does not allow to obtain a representative profile of the different neoplastic cell subpopulations, their pattern of invasion and related risk factors 14. In fact, distinct areas of OSCC may express a diverse array of features, thus preventing a comprehensive tumor characterization through an incisional biopsy alone 15.

miRNAs are small non-coding RNA molecules, which show remarkable stability in biologic fluids and altered expression in various cancers, both promising characteristics for the diagnostic and prognostic definition of tumors by means of a liquid biopsy. While the efficacy of some serum miRNAs as OSCC diagnostic and/or prognostic biomarkers have already been tested 16,17, studies on salivary miRNAs in OSCC are still scarce and mostly focused on validation of predetermined biomarkers identified in tissue analysis 18, further increasing the risk of bias and methodological inconsistencies.

In this study, we analyzed global salivary miRNA expression in a cohort of OSCC patients and healthy controls, with the aim to define its diagnostic and prognostic potentials and to provide a preoperative identification of high-risk tumors, regardless of conventional staging and risk factor stratification.

## Methods

### Patient cohort

The present study was performed following the Declaration of Helsinki set of principles and approved by the Research Review Board - the Ethic Committee - of the Spedali Civili, Brescia, Italy (study reference number: NP1545). Written informed consent was obtained from all participants. Saliva samples were collected from consecutive patients with a diagnosis of primary OSCC, which were referred for primary treatment to the Unit of Otorhinolaryngology-Head and Neck Surgery of the Spedali Civili-University of Brescia (Italy), between October 2013 and September 2016 (training set) and between January 2018 and February 2020 (test set). All patients were staged according to the 8th Edition of the AJCC-UICC TNM system. Normal saliva samples were obtained from patients with no oral lesions and matched with OSCC patients regarding smoking and alcohol consumption.

### Saliva collection

Patients were requested to refrain from smoking, eating, drinking and oral hygiene procedures for at least 1 h prior to saliva spitting. Unstimulated whole saliva was collected after mouth rinsing with 10 mL of sterile PBS and immediately processed by centrifugation at 2600 g for 15 min at 4°C to remove cellular components, according to a previously published protocol 19. Saliva supernatant was divided into 250-µl aliquots and stored at -80°C until further miRNA extraction.

### RNA isolation

Total RNA was extracted from 200 µl of saliva using miRNeasy Mini kit (Qiagen, Hilden, Germany), with a modified protocol for co-purification of small RNAs from liquid samples according to Todeschini *et al.* 20.

Ten synthetic spike-in RNA oligos without sequence homology to known human miRNAs were added to samples to control for variations during the preparation of total RNA and subsequent steps, as previously described 20.

### miRNA profiling by microarray

Global profiling of salivary miRNAs was carried out using commercially available G4872A-046064 human miRNA microarray (Agilent Technologies, Santa Clara, CA, USA), customized with probes for the detection of specific RNA oligo spike-in. Fifteen µl of total RNA were dried by speedvac and resuspended in 2 µl H_2_O before being labelled and hybridized on miRNA array, according to miRNA labelling and hybridization kit instructions (Agilent Technologies). Following washing and scanning with laser confocal scanner (G2565BA, Agilent Technologies), miRNA microarrays underwent standard post-hybridization processing and the intensity of fluorescence was calculated by Feature Extraction software version 11 (Agilent Technologies). Raw data are available at GEO database (accession number pending).

### miRNA quantification by quantitative real-time PCR

The miRCURY locked-nucleic acid Universal real-time (RT) miRNA PCR system (Exiqon, Qiagen Hilden, Germany), based on universal reverse transcription followed by RT PCR amplification with miRNA-specific primers (Supplementary Table S1), was used for first-strand cDNA synthesis and SYBR Green-based amplification.

In total, 4 µl of total RNA were used as input for reverse transcription following manufacturer's instructions. A total of 4 µl of a 50X dilution of cDNA were used for subsequent qPCR experiments, run in triplicate using Bio-Rad CFX96 Real-Time system. An inter-run calibration sample was used in all plates to correct for the technical variance between the different runs and to compare results from different plates. The 2^-∆∆Cq^ method was used to analyze raw Cq data following normalization with selected reference miRNAs and results were displayed as relative expression.

### miRNA normalization strategy

To accurately quantify miRNA levels and compare them between samples, a proper normalization relative to an endogenous miRNA is mandatory 21. In the absence of established endogenous miRNAs for reliable expression data normalization, we selected potential candidates according to their use in published studies, carrying out a Medline search using the MeSH terms “salivary miRNA” and “oral cancer” and “real-time PCR”.

### Validation on external database (TCGA dataset analysis)

Level 3 miRNA sequencing data (RNA-Seq) for the Head and Neck Squamous Cell Carcinoma Tissues (TCGA-HNSC project) were downloaded from GDC Data Portal (NIH) using GDC queries (harmonized dataset). For each sample, mapped reads per millions (RPM) were sum up across unique isoform IDs. Only samples annotated as oral squamous cell carcinomas with matched primary solid tumor (code 01) and normal solid tissue (code 11) were retained. Additional filter criteria were applied to tissue/organ of origin to ensure the comparability of the two collected cohorts. A total of 14 matched tumor-normal samples were selected for further analysis (TCGA code: HD-8635, CV-6961, CV-6959, CV-6939, CV-6933, CV-7238, CV-7235, CV-6936, CV-6934, CV-7103, CV-6956, CV-7255, HD-A6HZ, CV-7438).

### Statistical analysis

All statistical analyses were performed using R software (version 3.4).

#### Pre-processing and differential expression analysis of microarray data

Salivary raw microarray data comprised 1361 miRNAs repeated 40-times. The expressions of the 40 miRNAs replicates were used as technical replicates and quality control. For pre-processing and normalization steps, we follow the strategy proposed in Todeschini *et al.* 20. Briefly, we selected only miRNAs with at least 75% of samples -either within patients or healthy controls - with more than 20% good quality replicates (using gisPosandSig Agilent flag). The miRNA replicates within samples were summarized using mean values. The few missing values still present (0.9%) were imputed with k-nearest neighborhood method. Expression data of the remaining 826 miRNAs were normalized using cyclic LOWESS algorithm, in which the ten spikes in oligos were used as stabilizing factors (weight = 10). Empirical Bayes test (limma package) was used for differential expression analysis and Benjamini-Hochberg correction was applied for multiple testing correction.

#### RT-qPCR data and equivalence analysis

Normalized RT-qPCR data were obtained as the difference between Cq values of target miRNA and the selected reference (∆Cq); in case of multiple references, the arithmetic mean computed by the sample was used. Batch effect and age bias were removed from Cq values using the beta coefficients of the linear regression model estimated for each miRNA. Normalized Ct values in different groups were compared, performing one-tail paired sample t-test (OSCC versus healthy; pre- versus post-operative saliva). The equivalence of reference candidates was assessed using both Normfinder approach 22 (R package, version 5) and the two-one-sided t-tests procedure 23 (equivalence, R package) with the adoption of the default confidence level (alpha=0.05). To ensure an adequate power of the test the equivalence ranges were chosen depending on data variability following Wellek criteria 24.

#### ROC curves

The Receiver Operating Characteristic (ROC) curves were used to assess diagnostic performances of each miRNA using normalized Ct values. Logistic multivariate model was applied to test the diagnostic performance of the integration of all miRNAs. The numeric value of the area under the ROC curve (AUC) and its confidence interval was calculated with 20 repeated 5-folds cross validation estimates (cvAUC, R package). Each cross-validation fold was stratified by phenotype (OSCC/healthy individual).

#### Survival analysis

Disease-free survival (DFS) was defined as the time from the first surgery to the date of the first recurrence or last follow-up. The association of miRNA profiles and DFS was assessed based on the Cox proportional hazard model, with both univariate and multivariate analysis.

#### Pathway analysis

miR-423-5p/gene interactions were obtained from the manually curated database DIANA-TarBase (versione 7.0) via web server interfaces 25. In order to assess whether a certain biological pathway was significantly enriched, we performed an Over-Representation Analysis (ORA) based on the hypergeometric test (Benjamini-Hochberg correction), as proposed by Backes *et al.* 26. KEGG pathway annotations were retrieved using graphite package (version 1.34.0) 27.

## Results

### Discovery of potential salivary miRNA biomarkers by microarray

A total of 147 subjects were enrolled in the study, including 58 healthy individuals and 89 OSCC patients, divided into a training set and a test set according to the study design (Figure S1). Clinicopathological characteristics are summarized in Table 1. In the discovery stage, we performed global miRNA expression profile on preoperative saliva from 50 patients with primary OSCC and 42 healthy controls (training set), with the aim of identifying miRNAs both differentially expressed between OSCC patients and healthy subjects and characterized by the potential of predicting prognosis, in terms of DFS in OSCC patients.

**Figure 1:**
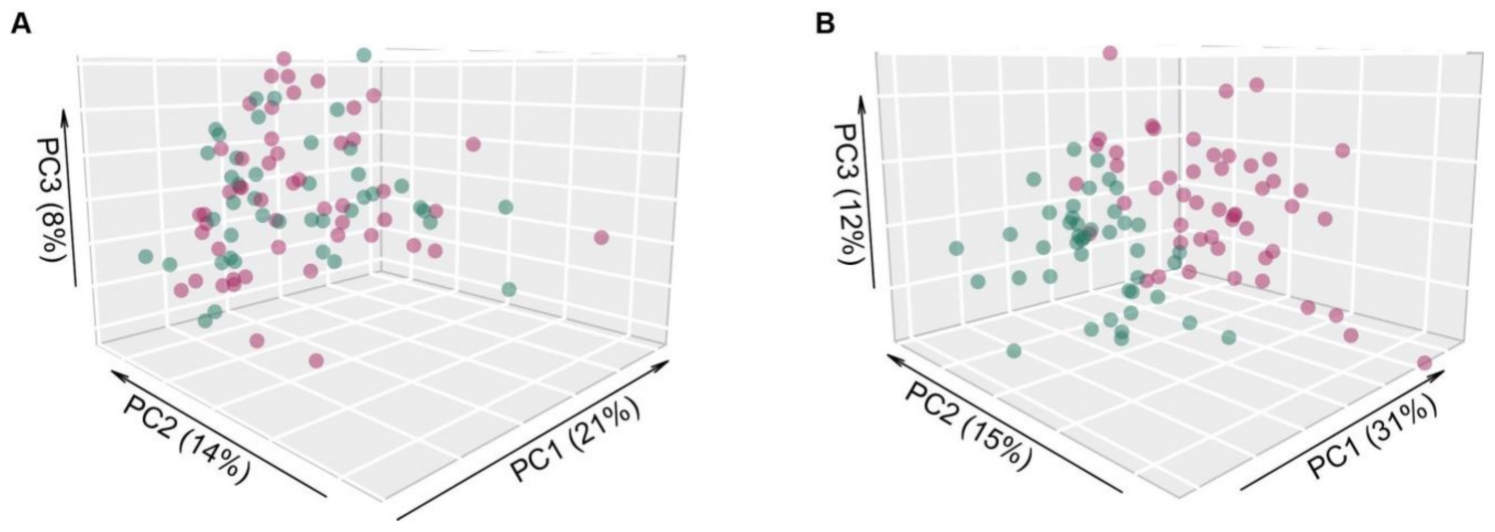
** Principal component analysis of microarray expression profiles.** Scatter plot of the first three principal components using (**A**) the entire collection of miRNAs profiles (n=826) and (**B**) only the differentially expressed ones (n=25), in OSCC patients (red dots) and healthy donors (green dots). The percentage of explained variance for each principal component is reported in brackets.

Salivary raw microarray data comprised 1361 miRNAs. Among them, 826 miRNAs fulfilled the filtering criteria described in the method section and resulted as detected in saliva samples. In total, 25 out of 826 miRNAs (3%) were identified to be differentially expressed (adjusted p ≤0.05) between saliva of OSCC patients and healthy controls (Table 2), indicating a distinct salivary miRNA profile in OSCC patients (Figure 1). Twenty out of 25 miRNAs (80%) were significantly upregulated in saliva samples of OSCC patients compared to controls, whereas five miRNAs (20%) were downregulated.

To further discriminate potential prognosticator candidates among the differentially expressed miRNAs, we evaluated survival association, considering DFS as the endpoint. In univariate survival analysis, seven out of 25 miRNAs were significantly associated with DFS with a relaxed threshold of 0.1 (Table 2).

### Validation of candidate salivary miRNAs by RT-qPCR

Following microarray data analysis, a set of potential miRNAs was chosen for validation by RT-qPCR, based on the following criteria: i) high expression level (log2 average expression ≥6); ii) commercially available and experimentally validated locked-nucleic acid PCR primer set; and iii) statistical significance in both the comparison between saliva of OSCC patients and healthy controls (≤0.05) and univariate survival analysis (≤0.1).

Four miRNAs (miR-106b-5p, miR-320e, miR-423-5p and miR-193b-3p) were selected according to the defined criteria, as shown in Table 2. Their expression was evaluated by RT-qPCR in a cohort of 55 OSCC patients and 39 healthy controls from the training set, including 48 and 37 microarray-profiled samples, respectively.

A median Cq <30 was considered indicative of a reliable detection regarding circulating miRNA quantification, as reported by other groups 28. MiR-106b-5p, miR-423-5p and miR-193b-3p were confirmed to be expressed at high levels (median^Cq^ = 27.7, σ^Cq^ = 2.2; median^Cq^ = 28.9, σ^Cq^ = 1.9; median^Cq^ = 27.9, σ^Cq^ = 1.9, respectively) in almost all saliva samples (average detection of 99.5%). On the contrary, miR-320e exceeded the reliability threshold and was discarded from further analysis (median^Cq^=32.5, σ^Cq^=1.8).

### Selection of reference miRNAs for RT-qPCR data normalization

The systematic evaluation of published articles on the analysis of miRNA expression in salivary samples revealed three miRNAs reported to be stably expressed: miR-16-5p, miR-484 and miR-191-5p 19,29. The expression of these putative references was evaluated by RT-qPCR and tested for stability in the cohort of 55 OSCC and 39 healthy subjects. Since normalization by multiple references is recommended by MIQE guidelines 30, we have also considered all the possible combinations of the three references, as arithmetic mean across each sample.

According to the equivalence tests (Table 3), a normalization factor based on the average expression levels of the three miRNAs was the most significantly equivalent between normal and malignant samples (p=0.004). Interestingly, although miR-191-5p individually showed a borderline equivalence (p =0.105), it is required to enhance the overall stability of the normalization factor. All these findings were confirmed with NormFinder (Table 3) and are in accordance with the literature 19,29.

Importantly, the same normalization factor showed to be prognostically stable within the cohort of OSCC patients (p=0.016), since the estimated hazard ratio (HR) for DFS was included in the equivalence margins (γ=0.5). These results indicate miR-484, miR-16-5p and miR-191-5p as suitable and robust normalizers for relative quantification in both diagnostic and prognostic studies in OSCC.

### 3-miRNA signature as non-invasive diagnostic biomarkers for OSCC patients

The arithmetic mean of miR-484, miR-16-5p and miR-191-5p was used to normalize miR-106b-5p, miR-423-5p and miR-193b-3p raw Cq data (Table 4).

**Figure 2:**
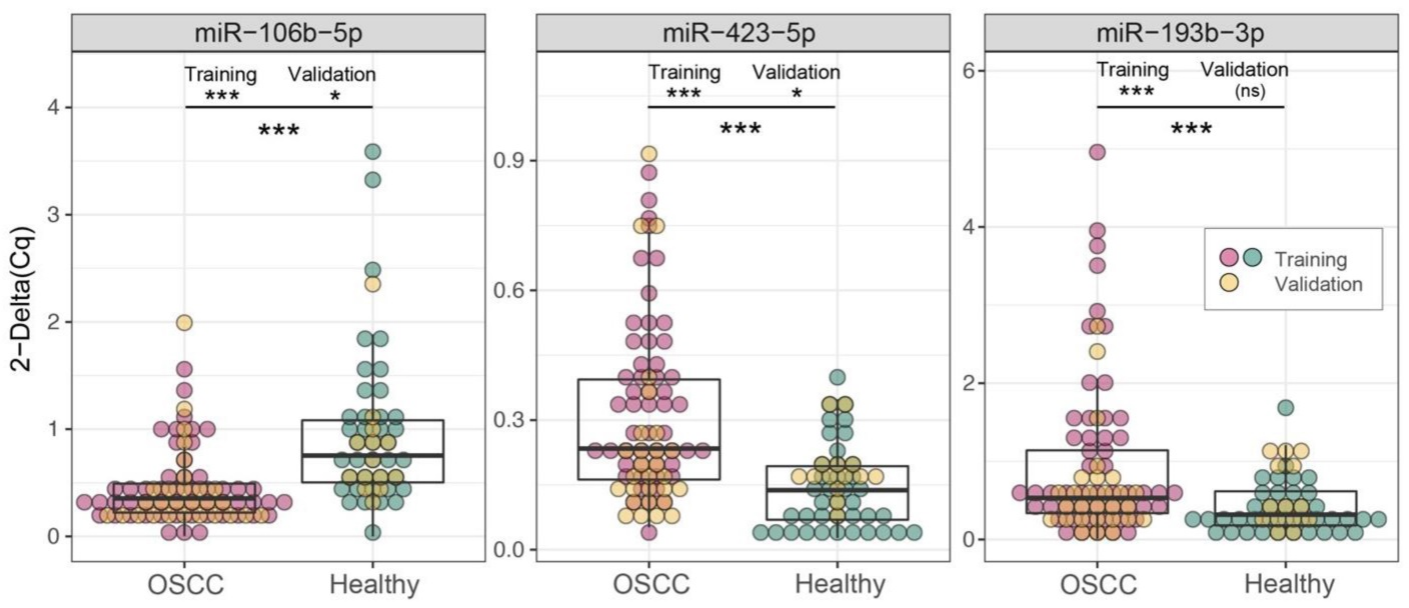
** Boxplots of the three candidate biomarkers evaluated by RT-qPCR in two cohorts.** Comparison of 2^-ΔCq^ values in OSCC patients (red/yellow dots) and healthy controls (green/yellow dots), in training (red/green dots), validation (yellow dots) and merged cohorts, (one-tails *t* test, ***p ≤ 0.001, **p ≤ 0.01, *p ≤ 0.05, ^*p ≤* 0.1 ). Complete results are reported in Table 4, Table 8 and Table S4, respectively.

All candidate miRNAs confirmed a significant differential expression in the saliva of OSCC patients compared with healthy controls (Table 4 and Figure 2), in accordance with results and trends of microarray data (Supplementary Figure S2).

**Figure 3:**
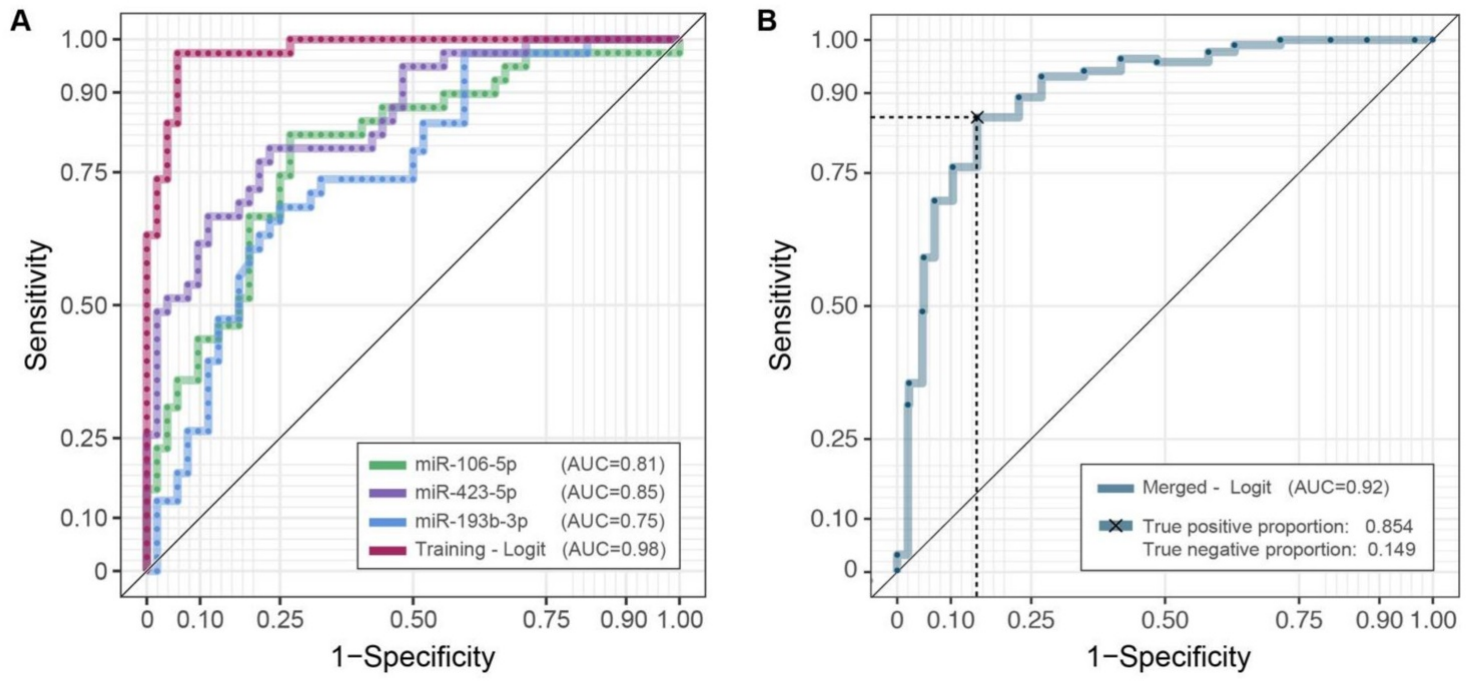
** ROC curves and AUC values.** Diagnostic performances of the three candidates miRNAs profiles (2^-ΔCq^) and their integration employing a logistic model in the training (**A**) and the merged cohorts (**B**). For the latter, the average of twenty repeated 5-fold cross-validated roc curves is depicted. Complete results are reported in Table S3.

To assess the accuracy of miR-106b-5p, miR-423-5p and miR-193b-3p as diagnostic biomarkers, we estimated ROC curves (Figure 3A), separately on each miRNA expression and on their integration, employing logistic model predictions (Supplementary Table S2). As reported in Table S3 the AUC values - based on repeated k-fold cross validation approach - were 0.813 (Se = 0.842; Sp = 0.731), 0.851 (Se = 0.885; Sp = 0.639), 0.748 (Se = 0.750; Sp = 0.639) and 0.98 (Se = 0.974; Sp = 0.942), respectively. The superior performance of the three-miRNA combination highlights its potential role as a diagnostic biomarker for OSCC detection.

### High miR-423-5p expression is an independent predictor of reduced DFS in OSCC patients

To ascertain whether miR-106b-5p, miR-423-5p and miR-193b-3p exhibited a potential as prognosticators, we conducted a survival analysis using each candidate miRNA normalized expression value (2^-ΔCq^), either alone in univariate analysis or in combination with the number of positive lymph nodes as covariate in multivariate analysis. As shown in Table 5, the number of positive lymph nodes (LNs) is the only feature associated with DFS in multivariate survival analysis, when considering clinical characteristics.

When LNs is added, together with the three candidate miRNAs, to the multivariate survival model (Table 6), miR-423-5p is the only showing independent prognostic potential (p = 0.027). Interestingly, when we replaced the absolute number of positive LNs with the three-group classification proposed by Ho and colleagues 31, the role of miR-423-5p became crucial for risk classification in the intermediate/extreme classes (Table 7). The reported evidence suggests the addition to this scheme of a level of stratification based on the miR-423-5p expression for patients with at least one positive LN.

**Figure 4:**
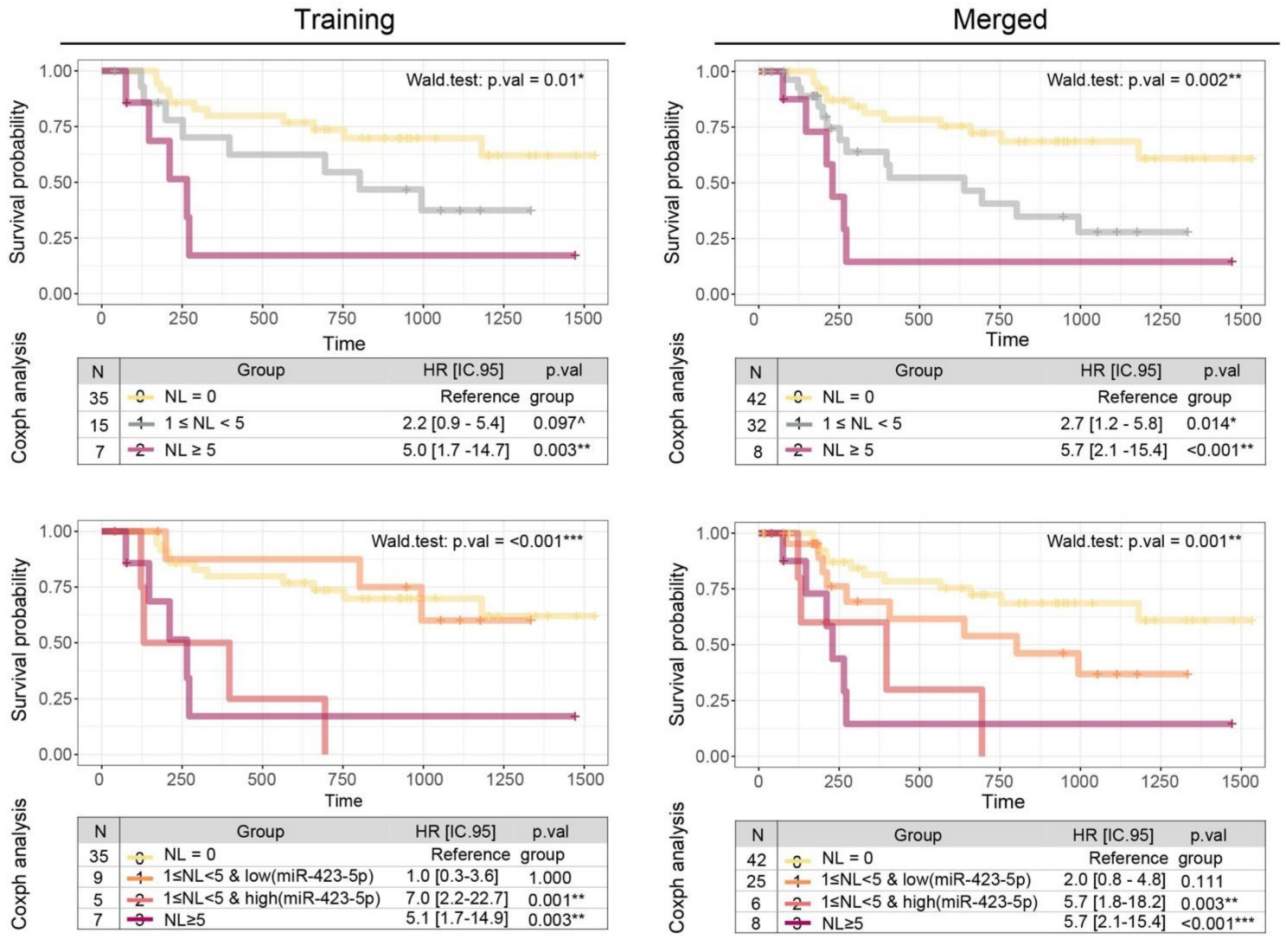
** Kaplan-Meier curves and Coxph survival analysis showing the prognostic potential of miR-423-5p.** Comparison of the prognostic value of the *three-groups* schema (upper panels) that account only for the number of positive lymph nodes (NL) and the *four-groups* model (bottom panels) that integrates miR-423-5p profile in intermediate risk group stratification, in the training (left panels) and merged coorts (right panels).

Due to the low sample size of the high-risk group (LN ≥ 5), we evaluated the miRNA integration only in the intermediate one (1 ≤ LN < 5), using a split strategy based on the mean level (cut.off = 0.49). Compared to the LN three-group classification (Figure 4a, p = 0.01), miR-423-5p expression improves the overall ability to predict DFS in OSCC patients (Figure 4b, *p <* 0.001): patients of the intermediate class with low levels of miR-423-5p exhibit a HR comparable to those with negative LNs; on the contrary, patients of the intermediate class with high levels of miR-423-5p are characterized by reduced DFS, consistent with those with five or more positive LNs.

### Diagnostic and prognostic performance of salivary miRNAs is confirmed in a second cohort of OSCC and healthy subjects

To ensure the robustness of the proposed biomarkers, we tested their diagnostic and prognostic value in a second cohort of independent individuals (hereinafter, the *validation cohort*), composed of 28 OSCC patients and 14 healthy subjects (Table 1). Cq values, after removal of batch effects, were normalized using the mean of the reference miRNAs (mean (Cq): OSCC=26.69, Healthy: 26.65 - equivalence p.val=0.016). Due to age biases in the control set (Healthy: [71-91] years; OSCC: [24-91] years), a further correction was required in the comparison between the two groups within this cohort.

The diagnostic performances of the miR-423-5p and miR-106b-5p were substantiated (Figure 2 and Table 8), contrary to miR-193b-3p (p.val = 0.144). However, these results arise from the lower statistical power of the validation set: repeating the comparison on the merged cohorts (Figure 2 and Table S4) all the three biomarkers achieve extremely high significance (p <0.001).

Combining the three-miRNAs signatures (Figure 3B), employing the logistic model together with the repeated k-fold cross-validation approach, we can effectively discriminate OSCC patients and healthy controls also in the extended cohort, with a robust AUC estimate equal to 0.923 and the associated sensitivity and specificity of 0.854 and 0.851, respectively (Table S3).

By adding new OSCC patients to the DFS analysis (Figure 4, left panels), we duplicated the observations in the intermediate-risk class (from 15 to 32 patients) which became significantly different from the group with no lymph nodes (HR=2.7, p.val = 0.014). Applying the cut-off outlined in the previous paragraph, only one out of 17 new patients fall into the high-risk intermediate class, while all the remaining are in the low-risk intermediate class. Overall, the integration of the miR-423-5p is established as useful in the classification strategy (Wald test: p.val = 0.001), proving the higher similarity of the intermediate class with low-miR expression (HR = 2.0, pval = 0.11) to the class with zero lymph nodes, and the high-miR expression (HR = 5.7, pval = 0.003) with the class with five or more lymph nodes.

### miR-423-5p is a tumor-specific prognostic biomarker for OSCC

**Figure 5:**
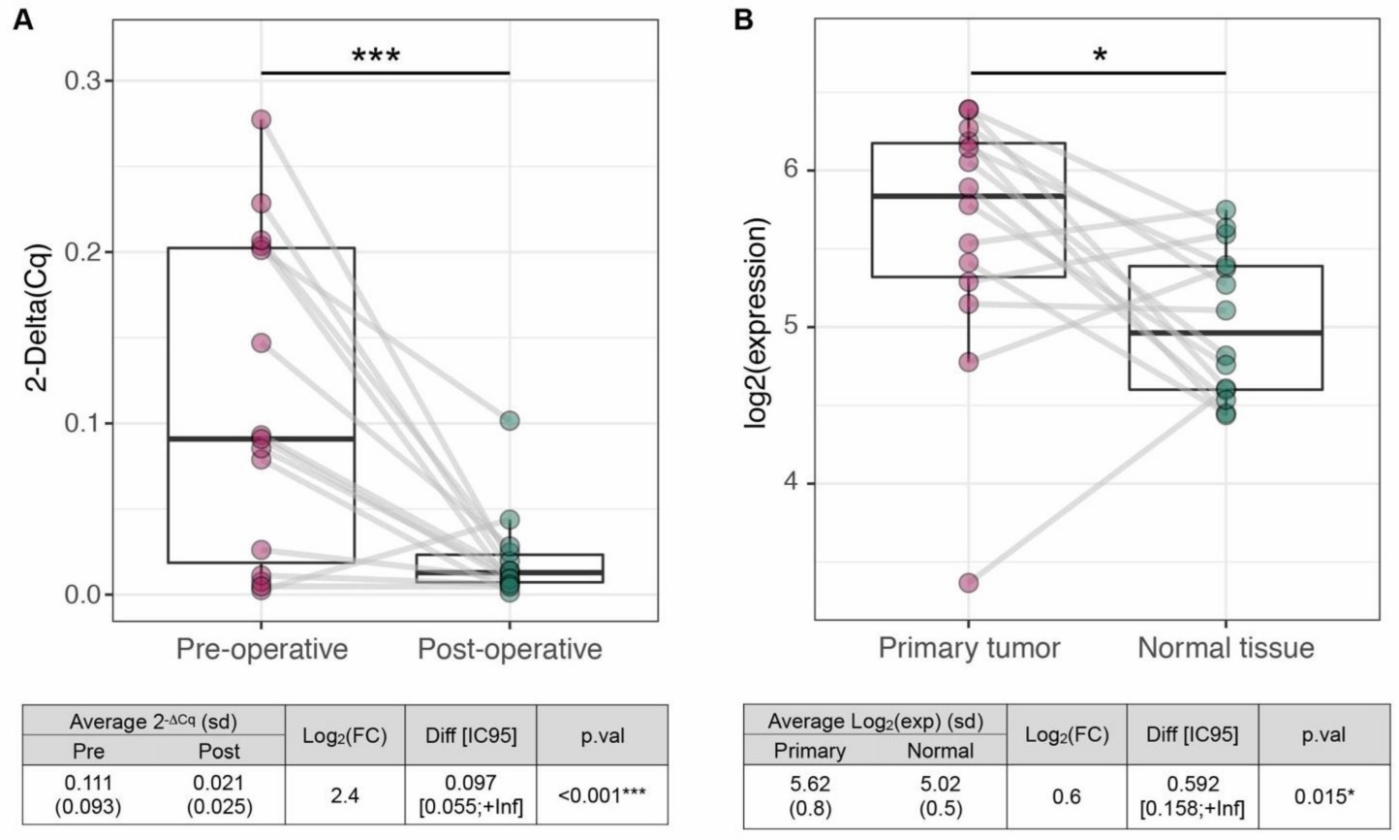
** Evaluation of mir-423-5p tumor specificity.** Comparison 2^-ΔCq^ values in paired pre- and post-operative samples (n=15) and (**B**) log2 expressions in tumors and matched normal tissues from TCGA collection (n=14), in OSCC patients (red dots) and healthy controls (green dots), (one-tail *t* test, ***p ≤ 0.001, *p ≤ 0.05).

To assess whether miR-423-5p has a tumor origin, we compared i) 15 pre- and post-operative saliva samples, coming from our OSCC cohort; and ii) 14 tumors and matched normal tissues, coming from TCGA collection. We observed a significant decrease of miR-423-5p salivary expression after tumor resection (LogFC=2.4, *p <* 0.001; Figure 5A) and its overexpression in tumor tissues compared to normal controls (LogFC=0.161, p=0.015; Figure 5B). Altogether, these results suggest that miR-423-5p, highly expressed in OSCC tissue and scarcely detected in saliva after tumor resection, may originate from cancer cells. To unravel possible functional roles of miR-423-5p, its putative target genes were included in KEGG pathway annotations to perform the Over-Representation Analysis (361 targets out of 6.229 annotated genes). 52 out of 302 pathways (17.2%) resulted as significantly enriched (adjusted p.val≤0.05, Supplementary File F1). These included several cancer-related pathways, such as cell signaling (MAPK, Notch), metabolism (N-Glycans biosynthesis) and those related to cell migration. Focal adhesion, cell junctions and gap junctions, in particular, were among the most enriched pathways, suggesting that miR-423-5p might play a role in OSCC progression and metastasis (since most of the biological functions of the target genes are involved in it).

## Discussion

In the conceptual framework of liquid biopsy, saliva has the potential to provide an overall view of OSCC tumor heterogeneity, thanks to its capability of collecting molecules originating from different cellular populations and neoplasm sites 32,33. Moreover, salivary analysis may offer significant advantages, since it requires a non-invasive procedure and can be performed in the preoperative period, without altering radiologic imaging quality, nor adding discomfort to the patients. Previous studies showed that saliva contains high levels of miRNAs that are protected by enzymatic degradation 34-36 and that their concentration is higher than in plasma, which might designate a higher sensitivity in detecting changes of expression 37.

In this study, first, we employed preoperative saliva samples from OSCC patients and healthy controls to investigate whether expression levels of specific miRNAs could be used as diagnostic biomarkers and, subsequently, we explored their impact on prognosis. This was performed using a standardized stepwise approach, through comprehensive evaluation of global miRNA expression in saliva, validation of candidate miRNAs, normalization by means of carefully selected stable endogenous controls and analysis of their diagnostic and prognostic potentials. Our results confirmed the promising value of three salivary miRNAs, miR-106b-5p, miR-423-5p and miR-193b-3p, which showed high and specific levels of expression in OSCC patients, with a remarkable diagnostic performance, potentially allowing detection of OSCC through salivary analysis alone. Importantly, we included in the present study an additional cohort of OSCC patients and healthy controls in which the diagnostic value of miR-106b-5p, miR-423-5p and miR-193b-3p have been evaluated by RT-qPCR and confirmed. Of note, miR-423-5p has been recently described in a plasma-based three-miRNA signature for early diagnosis of OSCC, confirming its overexpression in plasma and neoplastic tissue, as well as its involvement in various cancer pathways 38. In consideration of the relatively low incidence of OSCC in western countries, a highly effective diagnostic test could be regarded as a tool for secondary prevention (i.e., screening programs) only in at-risk populations (i.e., heavy smokers, alcohol abusers and patients with lesions at risk of malignant progression and/or an history of previous head and neck cancers). Moreover, patients previously treated for OSCC represent a particularly high-risk cohort that could significantly benefit from early diagnosis of recurrence. In fact, relapse frequently presents as locally advanced disease regardless of the follow-up policy, thus increasing the invasiveness of salvage treatment and reducing its effectiveness 39-41. In this view, this study could represent a first step towards validation of saliva-based screening methods that could help improving diagnosis in the initial stages of cancer recurrence.

Among clinical risk factors in OSCC, we confirmed in our series the critical prognostic role of the number of positive lymph nodes 31. This variable was considered in conjunction with salivary miRNA expression, identifying miR-423-5p as a significant prognosticator in multivariate analysis. Interestingly, miR-423-5p showed a complex and clinically-relevant interrelation with nodal status, allowing risk stratification of patients with 1-4 positive LNs, a subgroup comprising the vast majority of node-positive patients in OSCC. Patients with 1-4 positive LNs and low salivary miR-423-5p expression were classified as low risk, with a DFS comparable to that of node-negative patients. On the other hand, those with 1-4 positive LNs and a high salivary miR-423-5p expression were classified as high risk and presented a significantly lower DFS (comparable with that of patients with five or more positive LNs). Notably, those findings have been validated in a second cohort of OSCC patients, in which miR-423-5p expression was also confirmed to be useful in classifying patients in the intermediate-risk class.

This result is noteworthy considering that recent clinical reports highlighted the prominent independent prognostic role of positive LN number, surpassing even extranodal extension, and being proposed as a novel method for N stratification in the TNM system 31,42. In this context, salivary miR-423-5p may allow a further risk stratification that could lead to better treatment personalization, especially with regard to adjuvant therapy. To date, according to the National Comprehensive Cancer Network guidelines 3, postoperative radiotherapy is recommended in patients with minor adverse features (i.e., pT3 or pT4 primary, N2 or N3 nodal disease, nodal disease in levels IV or V, perineural invasion, vascular embolism, and lymphatic invasion); conversely, chemo-radiotherapy is generally suggested only in those presenting major risk factors (i.e., extranodal extension and positive margins). However, these criteria are only based on the results of two randomized clinical trials published in 2004, and further refinements may significantly improve indications for adjuvant treatment 43-45. In this regard, a retrospective observational cohort study using the National Cancer Database showed chemoradiotherapy having a survival benefit in comparison to radiation among patients with node-positive, resected, locally advanced head and neck squamous cell cancers, without pathologic major risk factors 46. It may be hypothesized that miR-423-5p may better define patient selection, thus improving survival and more precisely profiling adjuvant treatment.

Currently, the source of salivary miRNAs in cancer patients still remains undetermined. The present investigation demonstrates that our diagnostic and prognostic OSCC miRNAs have a tumor-associated origin, being significantly reduced after surgical resection and overexpressed in OSCC compared to normal tissues. A possible role of miR-423-5p as cancer biomarker to monitor OSCC patients during follow up for early recurrence may be supposed and deserves further investigations.

Analysis of salivary miRNAs in human cancer, including nasopharyngeal 47, pancreatic 48 and esophageal 29 tumors, has already been explored by several authors, providing invaluable preliminary data on the potential of miRNAs in saliva-based liquid biopsy. In OSCC, the majority of the studies focused on pre-established tumor-related miRNAs 37,49,50, while others included a genome-wide discovery phase that considered only a limited number of patients 19,34,51. We believe that a wider discovery approach by microarray on a large cohort of OSCC patients has enabled us to better characterize the entire salivary miRNA spectrum and its variability, providing a solid foundation for the subsequent selection process. Moreover, we performed a careful selection of reported reference salivary miRNAs in the literature and validated their stability in our experimental set with a stringent statistical approach, to fill the gap of knowledge regarding the lack of universally accepted reference for circulating miRNA RT-qPCR quantification. We ended up with the identification of three reliable reference miRNAs, used to normalize miR-106b-5p, miR-423-5p, and miR-193b-3p expression levels, thus obtaining the most accurate measure of expression and reducing the non-biological variation depending on pre-analytic and analytic variables. To our knowledge, this study represents the first identification of salivary reference miRNAs as reliable normalizers, validated for both diagnostic and prognostic purposes in OSCC patients.

In conclusion, by means of a high-throughput approach, followed by a stringent normalization and a strict validation process, our study identified a tumor-associated three-miRNA signature with diagnostic potential in OSCC and, for the first time, demonstrates that salivary miR-423-5p could assist risk stratification in OSCC patients. Of note, those findings have been validated and confirmed at the diagnostic and prognostic level on a second cohort of OSCC and healthy subjects.

Further investigations on independent cohorts of OSCC patients are warranted to confirm the diagnostic utility of our three-miRNA panel and the prognostic significance of miR-423-5p for application in the clinical setting.

## Supplementary Material

Supplementary figures and tables.

## Figures and Tables

**Table 1:** Clinicopathological features of OSCC patients and healthy donors.

Characteristics	Class		Training OSCC (n=61), n (%)	Training Healthy (n=44), n (%)	Validation OSCC (n=28), n (%)	Validation Healthy (n=14), n (%)
Age (years)	Mean [(range])		66.7 [(30-90])	50.72 [(22-92])	64.75 [24-91]	75.57 [71-91]
Gender	Female Male		18 (30)	16 (36)	9 (32)	4 (29)
			43 (70)	28 (64)	19 (68)	10 (71)
Smoke	No		25 (41)	23 (52)	14 (50)	4 (30)
	Yes NA		36 (59) -	21 (48) -	13 (46) 1 (4)	5 (35) 5 (35)
Alcohol	No		29 (47)	19 (43)	22 (78)	6 (43)
	Yes NA		32 (53) -	25 (57) -	5 (18) 1 (4)	6 (43) 2 (14)
N. of positive lymph nodes	Mean (range)		1.6 [0;13]	-	1.88 [0;7]	-
Stage (TNM)	pT	pT1	8 (13)	-	6 (21)	-
		pT2	15 (25)		6 (21)	
		pT3	25 (41)		8 (29)	
		pT4	9 (15)		8 (29)	
		NA	4 (6)		-	
	pN	pN0	36 (59)	-	11 (39)	-
		pN1	6 (10)		2 (7)	
		pN2	5 (8)		8 (29)	
		pN3	10 (17)		7 (25)	
		NA	4 (6)		-	
Extranodal Extension	No		10 (47.6)	-	9 (53)	-
	Yes		11 (52.4)		8 (47)	
Tumor site	Non-tongue	Floor of mouth	8 (13.1)	-	2 (7.1)	-
		Alveolar crest	14 (23)		6 (21.5)	
		Hard palate	4 (6.6)		2 (7.1)	
		Cheek	11 (18)		4 (14.3)	
	Tongue	Tongue	24 (39.3)	-	14 (50)	-
Tumor grade	G1		14 (23.0)	-	5 (17.9)	-
	G2		34 (55.7)		15 (53.6)	
	G3		12 (19.7)		8 (28.6)	
	NA		1 (1.6)		-	
Perineural invasion	No		31 (50.8)	-	7 (25)	-
	Yes		30 (49.2)		21 (75)	
Endovascular infiltration	No		48 (78.7)	-	14 (50)	-
	Yes		13 (21.3)		14 (50)	
Bone infiltration	No		50 (82.0)	-	19 (67.9)	-
	Yes		11 (18.0)		9 (32.1)	
Complementary therapy	No		26 (42.6)		11 (39.3)	
	Yes		34 (55.7)		15 (53.6)	
	NA		1 (1.6)		2 (7.1)	

**Table 2:** Panel of 25 differentially expressed miRNAs (Ebayes, *p ≤* 0.05) along with results of Coxph analysis for disease-free survival based on microarray log2 expression. MiRNAs that satisfied criteria for subsequent validation are highlighted (green). Number of samples: 92 (OSCC=50; Healthy=42).

miRNA	Average Log2(exp)	Diagnostic relevance (OSCC + healthy); Ebayes		Prognostic relevance (OSCC); DFS, Coxph	
		logFC (OSCC vs healthy)	Adjusted p-value	HR [95% CI]	p-value
miR-30c-5p	5.0	-0.632	<0.001***	0.7 [0.3-1.9]	0.473
miR-654-5p	5.6	0.970	<0.001***	1.2 [0.8-1.8]	0.424
miR-106b-5p	7.4	-0.705	0.001***	2.0 [1.0-3.8]	0.038*
miR-3680-3p	4.3	1.156	0.002**	0.7 [0.5-0.9]	0.011*
miR-320b	6.9	0.553	0.002**	1.7 [0.8-3.5]	0.133
miR-224-5p	4.9	0.988	0.002**	1.3 [0.8-1.9]	0.275
hsv2-miR-H9-5p	8.1	-1.846	0.002**	0.8 [0.7-1.0]	0.118
miR-320a	6.9	0.545	0.008**	0.6 [0.3-1.2]	0.120
miR-1290	12.1	1.091	0.008**	1.1 [0.8-1.7]	0.512
miR-1275	7.9	0.797	0.010**	0.9 [0.5-1.4]	0.536
miR-320c	8.9	0.736	0.010**	1.2 [0.8-1.8]	0.412
miR-30b-5p	5.7	-0.597	0.010**	0.8 [0.41.5]	0.473
miR-130a-3p	5.6	0.830	0.012*	1.5 [1.0-2.1]	0.039*
miR-3190-3p	4.3	0.888	0.014*	0.5 [0.3-0.7]	<0.001***
miR-3692-5p	4.6	0.729	0.014*	1.1 [0.7-1.5]	0.789
miR-26a-5p	7.2	-0.554	0.014*	0.9 [0.4-1.9]	0.709
kshv-miR-K12-7-5p	4.5	0.624	0.019*	1.0 [0.7-1.6]	0.833
miR-320e	7.0	0.483	0.023*	1.8 [1.0-3.4]	0.065^
miR-887-3p	4.2	0.519	0.023*	1.1 [0.7-1.9]	0.704
hsv1-miR-H16	5.1	0.661	0.024*	1.0 [0.6-1.7]	0.894
miR-320d	7.5	0.469	0.024*	1.4 [0.7-2.7]	0.341
miR-423-5p	6.4	0.425	0.026*	2.9 [1.1-7.9]	0.033*
miR-193b-3p	6.1	0.601	0.042*	1.9 [1.2-3.2]	0.007**
miR-205-5p	9.3	0.803	0.043*	1.3 [0.9-1.9]	0.168
miR-1246	14.0	0.801	0.046*	1.2 [0.8-1.7]	0.451

^≤0.1 *≤0.05 **≤0.01 ***≤0.001.

**Table 3:** Diagnostic and prognostic stability analysis (equivalence test, NormFinder) of three reference candidates and their possible combinations, based on RT-qPCR results. The most overall stable reference is highlighted (yellow). Labels: ①: miR-16-5p, ②: miR-484, ③: miR-191-5p. Number of samples: 94 (OSCC=55; Healthy=39).

Reference	Average Cq (SD)			Diagnostic stability (OSCC + healthy)				Prognostic stability (DFS) (OSCC)	
				*Equivalence Test^1^*		*NormFinder*		*Equivalence Test^2^*	
	NA	OSCC	Healthy	*Difference [95% CI]*	*p-value*	*Difference (SD)*	*Stability*	*log(HR) [95% CI]*	*p-value*
①	-	22.7 (1.9)	22.9 (1.7)	-0.20 [-0.82-0.43]	0.017*	0.19 (0.50)	0.18	-0.21 [-0.41 to -0.02]	0.007**
②	3	29.1 (1.8)	29.4 (1.4)	-0.30 [-0.86-0.25]	0.020*	0.32 (0.65)	0.26	-0.24 [-0.46 to -0.02]	0.024*
③	-	28.2 (2.0)	27.8 (1.9)	0.48 [1.17-0.11]	0.105^	0.51 (0.88)	0.34	-0.19 [-0.36 to -0.01]	0.002**
① + ②	3	25.9 (1.8)	26.1 (1.5)	-0.24 [-0.82-0.35]	0.017*	0.26 (0.40)	0.20	-0.23 [-0.44 to -0.02]	0.017*
① + ③	-	25.5 (1.9)	25.3 (1.7)	0.14 [-0.49-0.77]	0.013*	0.16 (0.26)	0.13	-0.22 [-0.41 to -0.02]	0.008**
② + ③	3	28.7 (1.8)	28.6 (1.6)	0.11 [-0.48-0.77]	0.007**	0.10 (0.16)	0.08	-0.23 [-0.44 to -0.01]	0.019*
① + ② + ③	3	26.7 (1.8)	26.7 (1.6)	0.02 [-0.58-0.61]	0.004**	0.00 (0.11)	0.02	-0.22 [-0.44 to -0.01]	0.016*

1Magnitude of similarity for difference between groups (ε) =1 2Magnitude of similarity for cox regression coefficient (γ) = 0.5 ^≤0.11 *≤0.05 **≤0.01 ***≤0.001

**Table 4:** Differential expressions analysis of the three candidate biomarkers evaluated by RT-qPCR, in the training cohort. Validated (*p ≤* 0.05) diagnostic miRNAs are highlighted (green). Number of samples: 94 (OSCC=55; Healthy=39).

miRNA	NA	Average 2^-ΔCq^		Log2(FC) OSCC vs healthy	T-test 1	
		*OSCC*	*Healthy*		*Diff [95% CI]*	*p-value*
miR-106b-5p	3	0.453	1.003	-1.15	-0.550 [-Inf to -0.330]	<0.001***
miR-423-5p	3	0.349	0.138	1.34	0.210 [0.157-Inf]	<0.001***
miR-193b-3p	4	1.085	0.382	1.51	0.740 [0.430-Inf]	<0.001***

1 Student's T-test, one tail. ^≤0.1 *≤0.05 **≤0.01 ***≤0.001

**Table 5:** Coxph survival analysis of clinicopathological features considered for disease-free survival. Features selected as covariate for the survival analysis of candidate prognostic miRNAs are highlighted (light blue). Number of samples: 57.

Feature (classes)	Number in classes			DFS - Univariate		DFS - Multivariate^1^	
	*0*	*1*	*NA*	*HR [95% CI]*	*p-value*	*HR [95% CI]*	*p-value*
Gender (female, male)	16	41	-	1.5 [0.6-3.9]	0.358	-	-
Smoke (no, yes)	18	39	-	0.5 [0.2-1.7]	0.114	-	-
Alcohol (no, yes)	26	31	-	1.6 [0.7-3.8]	0.230	-	-
Tumor site (not-tongue, tongue)	34	23	-	0.9 [0.4-2.2]	0.898	-	-
Tumor dimension (<4,=4)	25	32	-	2.6 [1.1-6.4]	0.032*	1.7 [0.6-4.5]	0.297
Complementary therapy (no, yes)	26	31	-	1.9 [0.8-4.4]	0.146	-	-
Tumor grade (G1 or G2, G3)	44	11	2	0.4 [0.1-1.5]	0.166	-	-
Age			-	1.0 [1.0-1.1]	0.255	-	-
N. of positive lymph nodes			-	1.3 [1.1-1.4]	<0.001***	1.2 [1.1-1.4]	0.003**

1Multivariate survival model accounted all features resulted significant (*≤0.05) in univariate model. ^≤0.1 *≤0.05 **≤0.01 ***≤0.001

**Table 6:** Coxph survival analysis for candidate prognostic miRNAs evaluated by RT-qPCR. Validated (*p ≤* 0.05) prognostic miRNAs are highlighted (green). Number of samples: 55. NL: number of positive lymph nodes.

miRNA	NA	Average 2^-ΔCq^ [range]	DFS - Univariate		DFS - Multivariate			
			*miRNA*		*miRNA*		*NL*	
			*HR [95% CI]*	*p-value*	*HR [95% CI]*	*p-value*	*HR [95% CI]*	*p-value*
miR-106b-5p	3	0.453 [0.004-1.540]	0.9 [0.2-3.5]	0.880	0.8 [0.2-3.0]	0.753	1.3 [1.1-1.4]	<0.001***
miR-423-5p^[a]^	3	0.349 [0.052-0.873]	1.2 [1.0-1.5]	0.102^	1.3 [1.0-1.6]	0.027*	1.3 [1.1-1.5]	<0.001***
miR-193b-3p	3	1.085 [0.007-4.959]	1.5 [0.8-1.6]	0.448	1.1 [0.7-1.7]	0.559	1.3 [1.1-1.4]	<0.001***

^≤0.11 *≤0.05 **≤0.01 ***≤0.001 ^[a]^ Confidence interval based on an increment of 0.1 unit of 2^-ΔCq^

**Table 7:** Interactions between miR-423-5p profile and the three classes of positive lymph nodes (NL) in disease-free survival prediction (Coxph). Number of samples: 55 (NA=3).

Variable	*HR [95% CI]*	*p-value*
NL=0	Reference group	
miR-423-5p^[a]^	1.11 [0.85-1.45]	0.431
(miR-423-5p^[a]^):(1≤NL<5)	1.25 [1.00-1.57]	0.048*
(miR-423-5p^[a]^):(NL≥5)	1.75 [1.21-2.52]	0.003**

^≤0.10 *≤0.05 **≤0.01 ***≤0.001 ^[a]^Confidence interval based on an increment of 0.1 unit of 2^-ΔCq^

**Table 8:** Differential expression analysis of the three candidate biomarkers evaluated by RT-qPCR, in the validation cohort. Validated (*p ≤* 0.05) diagnostic miRNAs are highlighted (green). Number of samples: 42 (OSCC=28; Healthy=14).

miRNA	NA	Average 2^-ΔCq^		Log2(FC) OSCC vs healthy	T-test 1	
		*OSCC*	*Healthy^2^*		*Diff [95% CI]*	*p-value*
miR-106b-5p	0	0.666	1.287	-0.95	-0.620 [-Inf to -0.188]	0.011*
miR-423-5p	0	0.147	0.101	0.54	0.050 [0.005-Inf]	0.033*
miR-193b-3p	0	0.225	0.174	0.38	0.052 [-0.029-Inf]	0.144

1 Student's T-test, one tail. 2 Corrected by batch-effect and age. ^≤0.1 *≤0.05 **≤0.01 ***≤0.001

## Abbreviations

AUC: area under the ROC curve
Se: sensitivity
Sp: specificity
DFS: disease-free survival
HR: hazard ratio
LNs: lymph nodes
OSCC: oral squamous cell carcinoma
ROC: receiver operating characteristic
RPM: reads per millions
RT-qPCR: quantitative real-time PCR
