# Supplementary material for: Genome-wide study of salivary miRNAs identifies miR-423-5p as promising diagnostic and prognostic biomarker in oral squamous cell carcinoma
Source: Theranostics. 2021 Jan 1;11(6):2987–99. doi: 10.7150/thno.45157 (PMC7806472; doi:10.7150/thno.45157)

Supplementary Tables

Table S1. miRCURY LNA^{TM} miRNA PCR assay IDs.

name	miRCURY LNA^{TM} miRNA PCR assay ID
hsa-miR-106b-5p	YP00205884
hsa-miR-423-5p	YP00205624
hsa-miR-193b-3p	YP00204226
hsa-miR-320e	YP02103235
hsa-miR-16-5p	YP00205702
hsa-miR-484	YP00205636
hsa-miR-191-5p	YP00204306

Table S2. Multivariate logistic regression for validated diagnostic miRNAs.

Generalized linear model accounted for 2-ΔCq values of the three diagnostic candidates, with group 0/1 (OSCC/Healthy) as response variable.

Variable	Coef (SD)	p-value
Intercept	1.523 (0.079)	<0.001***
miR-106b-5p	0.359 (0.062)	<0.001***
miR-423-5p	-1.005 (0.228)	<0.001***
miR-193b-3p	-0.114 (0.047)	0.018 *

^≤0.1 *≤0.05 **≤0.01 ***≤0.001

Table S3. Diagnostic performances based on ROC curves of the three candidate biomarkers.

**Model**	*AUC [95% CI][1]*	*False positive fraction (FPF)*	*True positive fraction (TPF)*	*Threshold[2]*
miR-106b-5p	0.813 [0.793 – 0.832]	0.269	0.842	0.462
miR-423-5p	0.851 [0.833 – 0.868]	0.316	0.885	0.161
miR-193b-3p	0.748 [0.725 – 0.770]	0.316	0.750	0.384
Training - Logit	0.980 [0.974 – 0.985]	0.058	0.974	0.484
Merged – Logit	0.923 [0.908 – 0.938]	0.149	0.854	0.437

[1] Based on 20 repeated 5-fold cross-validation estimates.

[2] Based on the farthest point from the diagonal.

Table S4. Differential expression analysis of the three candidate biomarkers, evaluated by RT-qPCR, in the merged cohort.

Validated ($p \leq 0.05$) diagnostic miRNAs are highlighted (green). Number of samples: 143 (OSCC=85; Healthy=58).

**miRNA**	**NA**	**Average $2^{-\Delta Cq}$**		**Log2(FC)**	**T-test [1]**	
		OSCC	***Healthy[2,3]***	**OSCC vs healthy**	***Diff [95% CI]***	***p-value***
miR-106b-5p	7	0.453	0.950	-1.07	-0.498 [-Inf to -0.322]	<0.001***
miR-423-5p	7	0.314	0.151	1.05	0.163 [0.118–Inf]	<0.001***
miR-193b-3p	8	0.930	0.435	1.09	0.495 [0.294–Inf]	<0.001***

[1] Student's T-test, one tail. [2] Corrected by batch-effect

^≤0.1 *≤0.05 **≤0.01 ***≤0.001

Supplementary Figures

Figure S1. Workflow of the study.

	Cohort	
	‣ 92 OSCC<br>‣ 58 Healthy patients	
Discovery	‣ 50 OSCC<br>‣ 42 Healthy patients	**Micorarry**<br>• Filtering: 826 miRNAs (out of 1.361)<br>• Normalization (cycle lowess): 10 spike-in oligos<br>• Differential expression (Ebayes, p.val≤0.05): 25 miRNAs (out of 826)<br>• DFS association (Coxph, p.val≤0.1): 7 miRNAs (out of 25)
Training	‣ 55 OSCC<br>‣ 39 Healthy patients	**RT-qPCR**<br>• Filtering: 4 miRNAs (out of 7)<br>• Quality control (median Ct<30): 3 miRNAs (out of 4)<br>• Normalization (TOST test, NormFinder, p.val≤0.05): 3 stably expressed miRNAs<br>• Differential expression (T-test one tail, p.val≤0.05): 3 miRNAs (out of 3)<br>• DFS association (Coxph, p.val≤0.05): 1 miRNAs (out of 3)
Validation	‣ 28 OSCC<br>‣ 14 Healthy patients	**RT-qPCR**<br>• Differential expression (T-test one tail, p.val≤0.05): 2 miRNAs<br>• Diagnostic potential (10 repeated 5-fold cross-validated roc curves, logit): AUC = 0.923<br>• Prognostic (DFS) potential (Coxph, p.val≤0.05): 4-groups schema
Pre-Post operative	‣ 15 OSCC (matched)	**RT-qPCR**<br>• Normalization (equivalence test): 3 stably expressed miRNAs<br>• Differential expression (paired T-test, one tail): 1 miRNAs

Figure S2. Boxplots of the three candidate biomarkers evaluated by microarray.

Comparison of log2 expression profiles in OSCC patients (red dots) and healthy controls (green dots), (Ebayes test, ***$p \leq 0.001$; *$p \leq 0.05$).

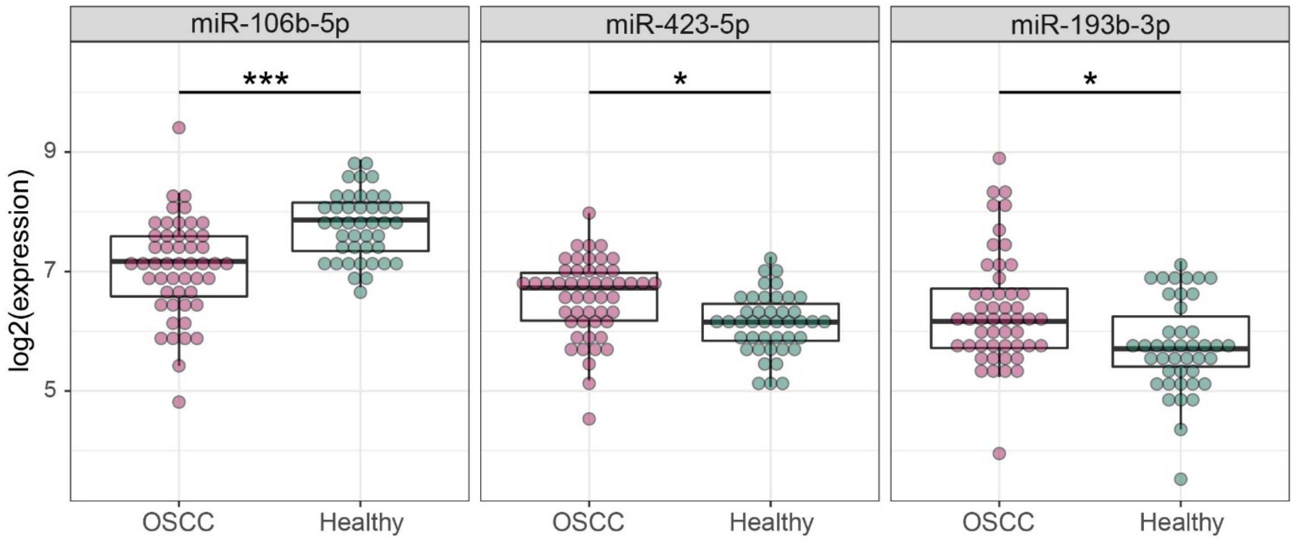

Supplement: Supplementary file 1 — Supplementary figures and tables. [file thnov11p2987s1.pdf]
